# Comparative analysis of mesenchymal stem cells derived from amniotic membrane, umbilical cord, and chorionic plate under serum-free condition

**DOI:** 10.1186/s13287-018-1104-x

**Published:** 2019-01-11

**Authors:** Jiao Ma, Jun Wu, Lei Han, Xiangxiang Jiang, Long Yan, Jie Hao, Hongmei Wang

**Affiliations:** 1 The State Key Laboratory of Stem Cell and Reproductive Biology, Institute of Zoology, Chinese Academy of Sciences, Beijing, 100101 China; 2 University of Chinese Academy of Sciences, Beijing, 100049 China; 3 Department of Obstetrics and Gynecology, The 306th Hospital of the Chinese People’s Liberation Army, Beijing, 100101 China

**Keywords:** Mesenchymal stem cells, Serum-free medium, Amniotic membrane, Umbilical cord, Chorionic plate, Trilineage differentiation efficiency, Gene expression pattern

## Abstract

**Background:**

Mesenchymal stem cells (MSCs) have emerged as a promising regenerative tool, owing mainly to their multi-differentiation potential and immunosuppressive capacity. When compared with MSCs classically derived from the adult bone marrow (BM), MSCs of neonatal origins exhibit superior proliferation ability, lower immunogenicity, and possible lower incorporated mutation; hence, they are considered as an alternative source for clinical use. Several researches have focused on the biological differences among some neonatal MSCs cultured in serum-containing medium (SCM). However, since it has been reported that MSCs possess different biological characteristics when cultured in serum-free medium (SFM), these comparative studies in SCM cannot exactly represent the results under the serum-free Good Manufacturing Practice (GMP) standard.

**Methods:**

Here, MSCs were isolated from three neonatal tissues, namely amniotic membrane (AM), umbilical cord (UC), and chorionic plate (CP), from the same donor, and their morphologies, immunophenotypes, trilineage differentiation potentials, global gene expression patterns, and proliferation abilities were systematically compared under chemical-defined SFM.

**Results:**

Our study demonstrated that these three neonatal MSCs exhibited a similar morphology and immunophenotypic pattern but various mesodermal differentiation potentials under SFM: amniotic membrane-derived MSCs showed a higher rate for osteogenic differentiation; chorionic plate-derived MSCs presented better adipogenic induction efficiency; and all these three neonatal MSCs exhibited similar chondrogenic potential. Moreover, by the analysis of global gene expression patterns, we speculated a possible higher proliferation ability of CP-MSCs in SFM, and we subsequently validated this conjecture.

**Conclusions:**

Collectively, these results suggest that MSCs of different neonatal origins possess different biological features in SFM and thus may represent an optimal choice for different clinical applications.

**Electronic supplementary material:**

The online version of this article (10.1186/s13287-018-1104-x) contains supplementary material, which is available to authorized users.

## Background

Mesenchymal stem cells (MSCs) are somatic stem cells which originate from mesoderm, and can differentiate into multi-lineages including adipocytes, osteocytes, chondrocytes, epithelial cells, neuron-like cells, and hepatocyte-like cells [1–3]. In addition, because of their low immunogenicity and capability to potently suppress or ameliorate immune responses [4], MSCs are considered as ideal candidates for therapeutic applications. After the first successful isolation from bone marrow (BM) in 1976 [5], MSCs have been subsequently isolated from a wide range of other tissues, such as adipose tissues, umbilical cord blood, placenta, skin, and hair follicles [6–9]. Over the past few years, MSCs derived from placentome tissues have attracted intensive attentions of more and more researchers [10], owing mainly to their noninvasive isolation methods, large-scale supply, and minimized ethical issues [11]. Moreover, it has been reported that mutations accumulate steadily over time and intrinsic mutational processes in adult stem cells can initiate tumorigenesis [12]. Hence, in comparison with those derived from adult BM or adipose tissues, MSCs derived from term placentome tissues can be immature cells with superior proliferation ability, lower immunogenicity [13], and possible lower incorporated mutation [14], which make them better options for clinical use. Different MSCs have been successively isolated from different layers of placentome tissues, including umbilical cord (UC), amniotic membrane (AM), chorionic plate (CP), chorionic villi (CV), and maternal decidua [15–18]. Considering the partly maternal origin of CV tissues [19], we thus focused on MSCs derived from the rest three neonatal tissues, namely amniotic membrane-derived MSCs (AM-MSCs), umbilical cord-derived MSCs (UC-MSCs), and chorionic plate-derived MSCs (CP-MSCs).

Meanwhile, as a heterogenous population of multi-potent stem cells with typical fibroblast-like morphology, MSCs of different tissue origins or culture conditions may exhibit diverse biological potentials [20]. Although AM-MSCs, UC-MSCs, and CP-MSCs share many more similarities and present even closer relations when compared with MSCs derived from adult tissues, it has been demonstrated that they also present different faces with each other. Wegmeyer et al. [15] reported that AM-MSCs and UC-MSCs showed different growth characteristics and distinct gene expression patterns. Kim et al. [18] reported that CP-MSCs possessed higher expression of adipogenesis-related genes but lower ability of mineralized matrix accumulation ability when compared with UC-MSCs. Araújo et al. [17] reported that MSCs of four neonatal sources (AM, UC, chorionic membrane, and placental decidua) presented relatively lower ability of adipogenesis but superior efficiency in osteogenesis. However, studies mentioned above paid their attention on neonatal MSCs cultured in serum-containing medium (SCM), which might bring uncertainties to the results owing to the appreciable batch-to-batch variation of serum. Even worse, the safety issues associated with animal or human serum can be never ignored, thus the utilization of SCM might thoroughly hinder the further clinical applications of these MSCs, due to a risk of the infectious pathogen contamination. Furthermore, it has been reported that human UC-MSCs cultured in serum-free medium (SFM) exhibited differently in growth rate, telomerase, and gene expression profile [21], which suggested that the comparative work performed in SCM cannot exactly represent the results in SFM.

Within this context, we designed the present study to systematically compare AM-MSCs, UC-MSCs, and CP-MSCs in chemical-defined SFM. These three MSCs were isolated from the placenta of the same donor, cultured in SFM; their morphologies, immunophenotypes, trilineage differentiation potentials, and proliferation abilities were compared and their different gene expression patterns were analyzed to evaluate their potential clinical applications in cell therapies in further studies.

## Methods

### Isolation and culture of AM-MSCs, UC-MSCs, and CP-MSCs

Healthy full-term human placental samples were collected according to the policy of the Ethics Committee of the 306th Hospital of the Chinese People’s Liberation Army, Beijing, China. Written informed consents were obtained from all donors before this study. Collected placentas were sterilely kept on ice and processed by explant methods within 4 h post-delivery. All the samples were used in accordance with standard experimental protocols approved by the Ethical Committee of Institute of Zoology, Chinese Academy of Sciences.

Briefly, UC tissues were cut into small sections, and the veins and arteries were clearly removed. Then, the AM and CP tissues were successively peeled from the human placenta. All the tissues were thoroughly washed with cold Dulbecco’s phosphate-buffered saline (DPBS; Gibco, Grand Island, NY, USA) and then separately cut into 0.5–1 mm^3^ small pieces. Minced small explants were transferred into 100-mm plates (Corning, NY, USA). A chemical-defined SFM (MSCGM-CD; Lonza, Walkersville, MD, USA) was carefully added. The plates were kept in 37 °C, 5% CO_2_ humidified atmosphere (Thermo Fisher Scientific, San Diego, CA, USA), and fresh medium was changed every other day. Colonies with fibroblast morphology usually appeared 10–14 days afterwards. At around 80% confluence, cells were detached using TrypLE™ Express (Invitrogen, Carlsbad, CA, USA) and then spilt at the ratio of 1: 3. Cells at passage 5 were utilized for all the further experiments.

### Flow cytometric analysis

The immunophenotype of MSCs was analyzed with the following antibodies: FITC-conjugated CD14, CD19, and CD45, and PE-conjugated CD34, CD73, CD90, CD105, and HLA-DR. Corresponding isotype-matched antibodies were used as controls. All the antibodies were purchased from BD Pharmingen (Franklin Lakes, NJ, USA). Cells were analyzed by flow cytometry using CytoFLEX (Beckman Coulter, Indianapolis, IN, USA). Data analysis was performed with CytExpert software (Beckman Coulter).

### Trilineage differentiation

Adipogenic, osteogenic, and chondrogenic differentiation experiments were performed following the instructions of human mesenchymal stem cell functional identification kit (R&D systems, Inc., Wiesbaden, Germany).

For adipogenic differentiation, MSCs were seeded into a 24-well plate at the density of 3.7 × 10^4^ cells/well, and maintained in culture medium until 100% confluency. Cells were then exposed to adipogenic differentiation medium for 3 weeks. Lipid droplets of the resultant differentiated cells were detected using Oil red staining (Sigma-Aldrich, St. Louis, MO, USA).

For osteogenic differentiation, 4.2 × 10^3^ cells were seeded per well. When cells reached 50–70% confluency, the medium was replaced with osteogenic differentiation medium and kept for 3 weeks. To assess osteogenic differentiation, Alizarin Red S staining (Sigma-Aldrich) was performed for the calcium-rich extracellular matrix.

For chondrogenic differentiation, 2.5 × 10^5^ cells resuspended in chondrogenic differentiation medium were centrifuged for 5 min at 200×*g* in a 15-mL conical tube (Corning). After 3 weeks, a chondrogenic pellet was harvest and fixed in 4% paraformaldehyde (PFA). Cryosection was performed and sections were stained with Alcian Blue (Sigma-Aldrich).

### RNA extraction and real-time PCR

Total RNA was isolated from MSCs using Trizol Reagent (Invitrogen) according to manufacturer’s instructions. Quality of RNA was controlled with NanoDrop 2000 spectrophotometer (Thermo Fisher Scientific). The cDNA was prepared by SuperScript™ II (Thermo Fisher Scientific) using 2 μg RNA.

To comparatively analyze the expression level of trilineage differentiation-related genes, the SYBR Green (TaKaRa, Dalian, China) detection method was employed and real-time quantitative PCR was performed on LightCycler 480 (Roche Applied Science, Indianapolis, IN, USA) with an annealing temperature of 60 °C using customized Real-Time ready 384 Panel (Roche Applied Science). The relative expression level of the differentiation-related genes in one particular induced MSC sample was normalized to 1, and the relative expression fold in other induced MSC samples was shown as 2^–ΔΔCt^. Real-time PCR was performed with samples from three independent donors, and each sample was tested in triplicate. Primers involved were listed in Additional file 1: Table S1.

### Transcriptome analysis

Four pairs of AM-MSC, UC-MSC, and CP-MSC RNA samples (12 samples in total) were sent to Annoroad Company for mRNA sequencing (RNA-Seq). The sequencing library for Illumina HiSeq 4000 sequencer was constructed with 0.2 μg of total RNA of each sample by PE150 strategy. Raw data and processed data were uploaded to the NCBI Gene Expression Omnibus database (accession number GSE118808). Data analysis was executed as previously reported [22]. Low expressed genes were removed. All 0 FPKM values were replaced by 0.01. We eventually identified 763 differentially expressed genes among these three MSCs based on twofold differences from 6637 genes. Unsupervised hierarchical clustering analysis was performed using Cluster 3.0 [23]. After the log-transformation of the input data, we then selected center genes with median. The adjusted data on genes and arrays were clustered using the average linkage method. Clustering results were presented and exported by TreeView 1.1.6r4 [23]. The principal component analysis (PCA) was made by R (3.5.0)/Bioconductor (3.7) with the “edgeR” and “limma” packages [24]. Kyoto Encyclopedia of Genes and Fenomes (KEGG) [25] and gene ontology–biological process (GO-BP) [26] enrichment analyses were performed using R/Bioconductor with the “clusterProfiler” package [27]. The Venn diagram was constructed at http://genevenn.sourceforge.net /.

### Colony-forming unit (CFU) assay

To assess the self-renewal capacity of MSCs, CFU-F efficiency assay was performed. 1 × 10^3^ viable cells at passage 5 were seeded in 100-mm plates (Corning). Following the cultivation for around 14 days (before colonies began to merge), the MSCs were washed with DPBS (Invitrogen), fixed with 4% PFA for 10 min and then stained with 1% toluidine blue (Sigma-Aldrich) solution for 30 min at room temperature. Stained colonies with at least 50 cells were counted for further analysis.

### Cell Counting Kit-8 (CCK8) assay

The experiments were performed following the instructions of Cell Counting Kit-8 (Sigma-Aldrich). MSCs of all three neonatal origins at passage 5 were utilized for the assay. Briefly, a total of 2 × 10^3^ viable cells were plated in each well of the 96-well plates (Corning). After the incubation for the first 24 h, the viable cell number was then tested every 24 h for seven consecutive days. To determine the number of viable MSCs, the optical density value at 450 nm was detected with Enspire™ Multimode Plate Reader (PerkinElmer, Baesweiler, Germany).

### Statistical analysis

Statistical analysis was performed using GraphPad Prism 5.0 software (Graph Pad Software, Inc., San Diego, CA, USA). All data were presented as the mean ± SEM. One-way ANOVA followed by Bonferroni multiple comparisons was utilized to determine the statistical significance. The result was considered of statistical significance when *p* < 0.05.

## Results

### AM-MSCs, UC-MSCs, and CP-MSCs exhibited similar morphology and immunophenotypic profiles

**Fig. 1:**
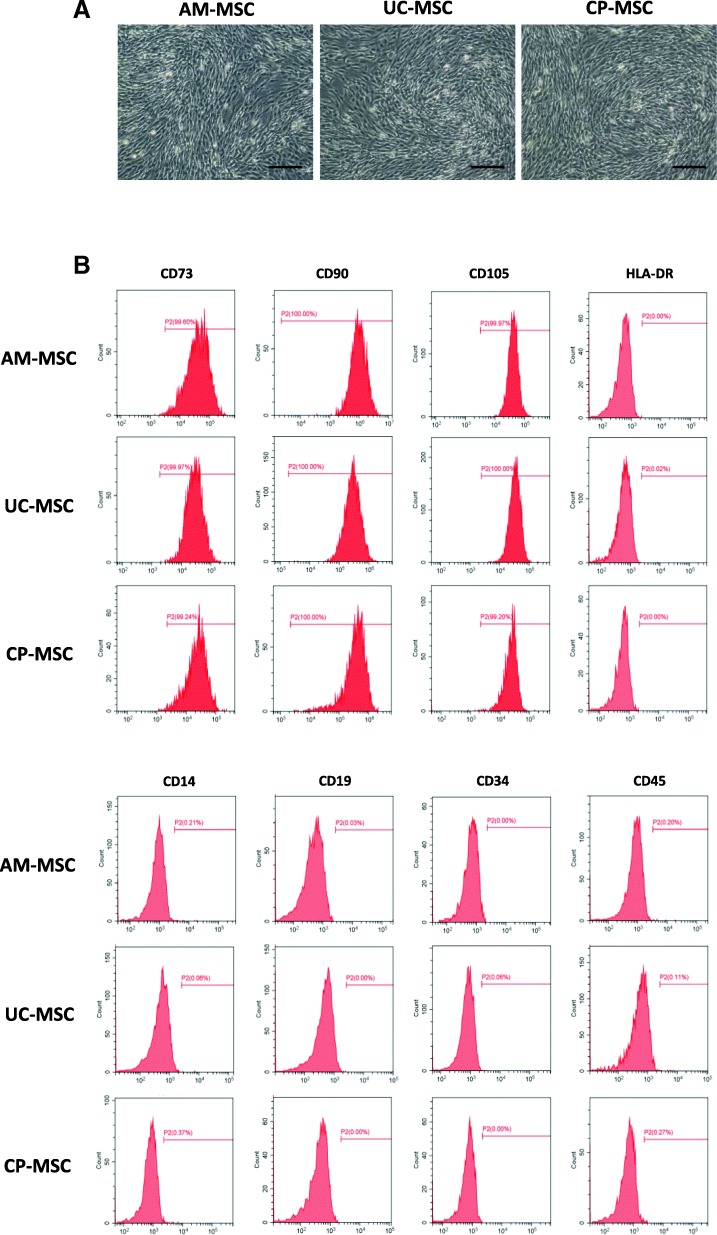
Morphology and immunophenotype of MSCs derived from AM, UC, and CP. **a** Phase-contrast microscope images of MSCs at passage 5. *Scale bar* = 200 μm. **b** Representative pictures of flow cytometric analysis of the surface marker expression on AM-MSCs, UC-MSCs and CP-MSCs

For the establishment of these three neonatal MSCs, healthy full-term placental samples were collected and processed within 4 h post-delivery. After the isolation of primary AM-MSCs, UC-MSCs, and CP-MSCs using explant methods, these MSCs were respectively expanded in the chemical-defined SFM. The morphologies of MSCs at passage 5 were assessed using inverted phase contrast microscopy. MSCs derived from all these three neonatal sources retained a fibroblast-like morphology and exhibited the spiral-shaped characteristics when reached confluence (Fig. 1a).

Flow cytometric analysis was then performed according to the MSC criteria proposed by the International Society for Cellular Therapies (ISCT) [1], which stipulated that the MSC population must express (≥ 95%) CD105, CD73, and CD90 but lack expression (≤ 2% positive) of CD45, CD34, CD14 or CD11b, CD79a or CD19, and HLA class II. MSC samples from three individual donors were analyzed, and our data demonstrated that all the three MSCs were negative for the MHC class II molecule HLA-DR, showed low expression of endothelial and hematopoietic markers (CD45, CD34, CD19, and CD14), and highly expressed typical MSC markers (CD73, CD90, and CD105) (Fig. 1b). It was revealed that there was no difference among these three MSCs in terms of immunophenotypic patterns (see Additional file 2: Figure S1).

### CP-MSCs exhibited superior adipogenic potential

**Fig. 2:**
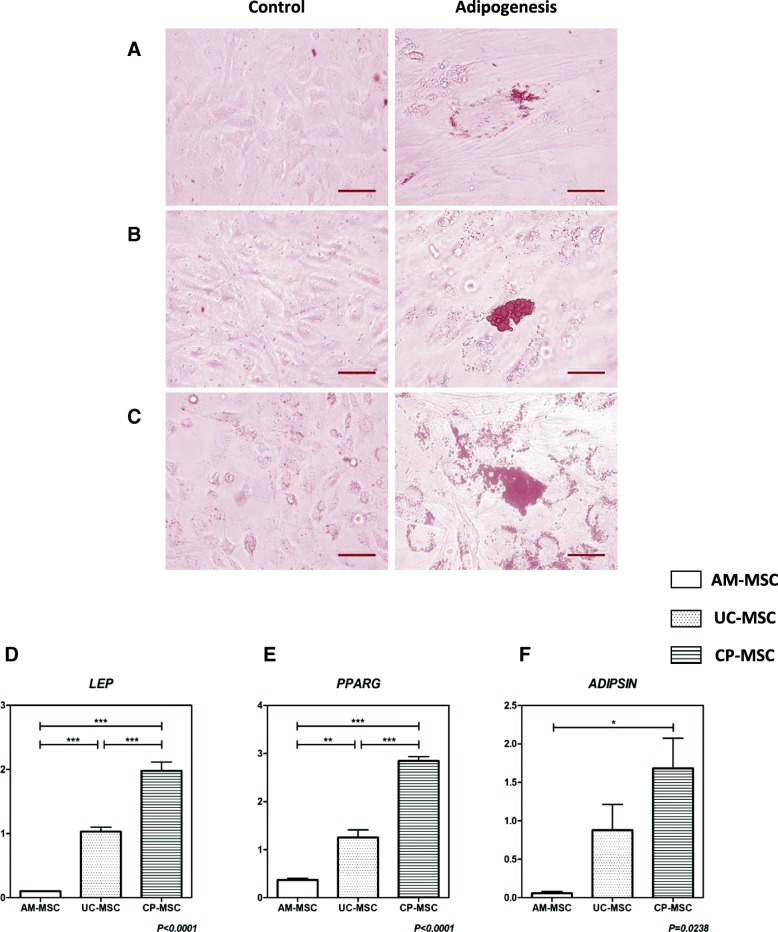
Adipogenesis of AM-MSCs, UC-MSCs, and CP-MSCs. **a–c** Representative pictures of three MSCs before and after the adipogenic differentiation were shown. The differentiation results of AM-MSCs (**a**), UC-MSCs (**b**), and CP-MSCs (**c**) were confirmed by Oil Red O staining. *Scale bar* = 50 μm. **d**–**f** Real-time PCR was performed to measure the relative expression levels of adipogenic differentiation related genes: *LEP* (**d**), *PPARG* (**e**), and *ADIPSIN* (**f**). All data were presented as the mean ± SEM (*n* = 3). Each sample was replicated in triplicates. *p < 0.05; **p < 0.01; ***p < 0.001

To test the adipogenic potentials of AM-MSCs, UC-MSCs, and CP-MSCs expanded in SFM, these three MSCs at passage 5 were cultured in the commercial adipogenic induction medium. At around 7 days post induction, MSCs turned to be flat and lipid vacuoles started to appear in the induced cells. After 21 days of induction, cells were fixed, and then the adipogenesis was verified by Oil Red O staining. The accumulation of cytoplasmic oil droplets could be distinctly observed in CP-MSCs while relatively weakly stained in UC-MSCs or AM-MSCs (Fig. 2a–c), indicating the superior adipogenic potential of CP-MSCs.

Real-time PCR was then performed to further confirm the adipogenic efficiency of these three MSCs. The quantification of *LEP*, *PPARG*, and *ADIPSIN* mRNA expression levels revealed that CP-MSCs exhibited the highest adipogenic efficiency (Fig. 2d–f), which was consistent with the results of Oil Red O staining. It was also shown that UC-MSCs expressed significantly higher *LEP* (Fig. 2d; *p* < 0.001) and *PPARG* (Fig. 2e; *p* < 0.01) than AM-MSCs, which indicated a superior adipogenic potential of UC-MSCs over AM-MSCs. Thus, it was concluded that among these three neonatal MSCs, the adipogenic potential was high in CP-MSCs, moderate in UC-MSCs, but relatively low in CP-MSCs.

### AM-MSCs showed superior osteogenic potential

**Fig. 3:**
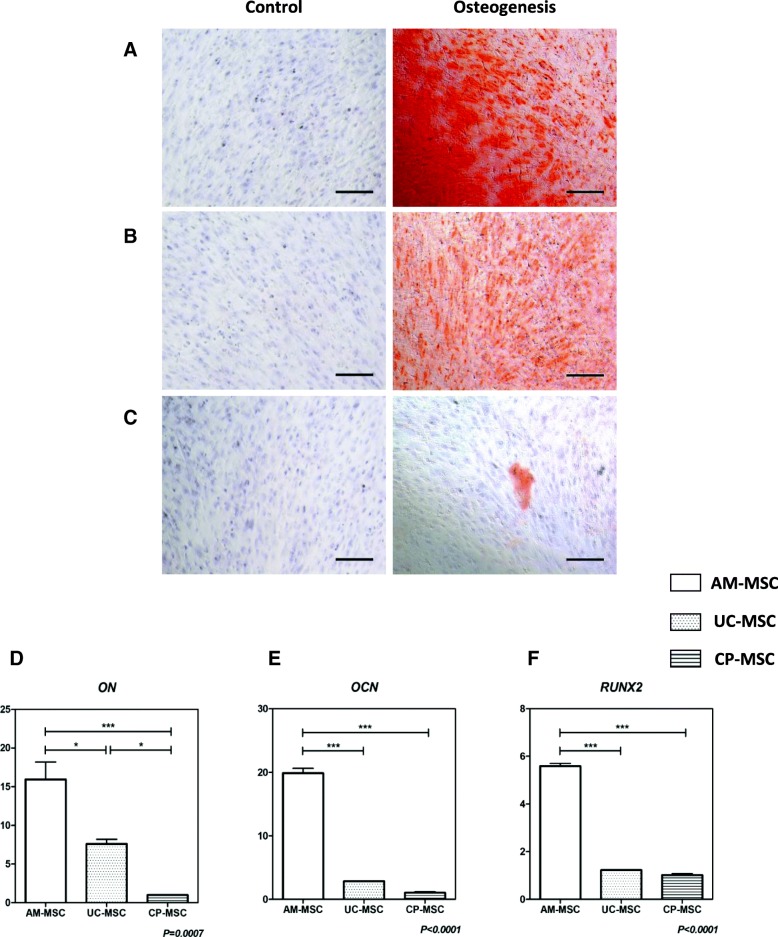
Osteogenesis of AM-MSCs, UC-MSCs, and CP-MSCs. **a–c** Representative pictures of three MSCs before and after the osteogenic differentiation were shown. The differentiation results of AM-MSCs (**a**), UC-MSCs (**b**), and CP-MSCs (**c**) were confirmed by Alizarin Red staining. *Scale bar* = 200 μm. **d**–**f** Real-time PCR was performed to measure the relative expression levels of osteogenic differentiation related genes: *ON* (**d**), *OCN* (**e**), and *RUNX2* (**f**). All data were presented as the mean ± SEM (*n* = 3). Each sample was replicated in triplicates. *p < 0.05; ***p < 0.001

To test the osteogenic potentials of AM-MSCs, UC-MSCs, and CP-MSCs, these three MSCs at passage 5 were cultured in the commercial osteogenic induction medium. After the incubation in osteogenic induction medium for around 21 days, Alizarin Red staining was performed when cells started to detach. The staining results confirmed that all the three MSCs underwent osteogenic differentiation, but with variable efficiencies. The red staining results indicating calcium deposit were very clear in AM-MSCs and moderate in UC-MSCs, while weak in CP-MSCs, which indicated a gradient descent of osteogenic potentials from AM-MSCs, UC-MSCs, to CP-MSCs (Fig. 3a–c).

The osteogenic capacity was then further evaluated by measuring the relative mRNA expression of related markers. According to the expression of *ON*, *OCN*, and *RUNX2*, expression level was three times higher in AM-MSCs than in CP-MSCs (Fig. 3d–e; *p* < 0.001). The expression level of *ON* showed a significant decrease from AM-MSCs to UC-MSCs and from UC-MSCs to CP-MSCs (Fig. 3d; *p* < 0.05). The quantification of *OCN* and *RUNX2* expression also showed to be three times higher in AM-MSCs than in UC-MSCs (3E-F; *p* < 0.001). Taken together with the Alizarin Red staining results, it was concluded that among these three neonatal MSCs, osteogenic efficiency was high in AM-MSCs, moderate in UC-MSCs, and low in CP-MSCs.

### AM-MSCs, UC-MSCs, and CP-MSCs displayed a similar chondrogenic potential

**Fig. 4:**
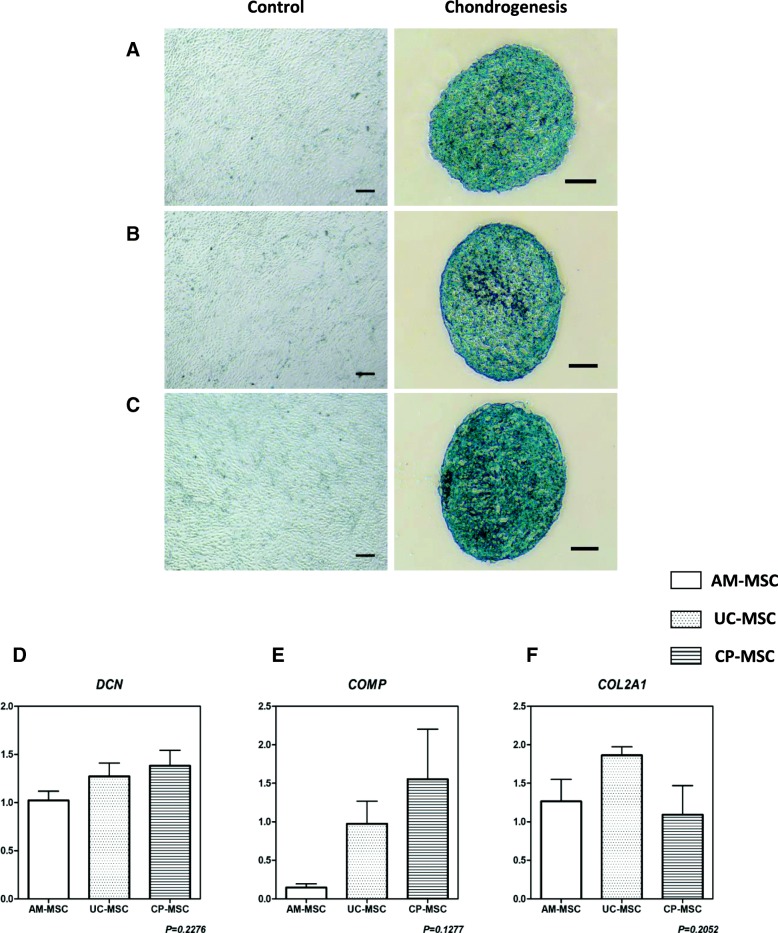
Chondrogenesis of AM-MSCs, UC-MSCs, and CP-MSCs. **a**–**c** Representative pictures of three MSCs before and after the chondrogenic differentiation were shown. The differentiation results of AM-MSCs (**a**), UC-MSCs (**b**), and CP-MSCs (**c**) were confirmed by Alcian Blue staining. *Scale bar* = 200 μm. **d**–**f** Real-time PCR was performed to measure the relative expression levels of chondrogenic differentiation related genes: *DCN* (**d**), *COMP* (**e**), and *COL2A1* (**f**). All data were presented as the mean ± SEM (*n* = 3). Each sample was replicated in triplicates

To test the chondrogenic potentials of AM-MSCs, UC-MSCs, and CP-MSCs expanded in SFM, these three MSCs at passage 5 were centrifuged in a 15-mL conical tube. After culture with the commercial chondrogenic induction medium for around 21 days, the chondrocyte pellets formed and were fixed and frozen sectioning was performed. The sections were then stained with Alcian Blue indicating cartilage proteoglycans. All tested MSCs exhibited positive staining results, and there was no obvious difference among AM-MSCs, UC-MSCs, and CP-MSCs (Fig. 4a–c).

Thus, real-time PCR was performed to further evaluate the chondrogenic efficiency. It was found that all these three neonatal MSCs showed a similar chondrogenic potential according to their expression of *DCN*, *COMP*, and *COL2A1* (Fig. 4d–f).

Collectively, the results indicated that MSCs derived from different neonatal tissues exhibited their own superiority to differentiate into different mesodermal lineages when cultured in SFM, suggesting a preferable option of AM-MSCs for osteogenesis and CP-MSCs for adipogenesis.

### Transcriptional differences among the three types of MSCs

**Fig. 5:**
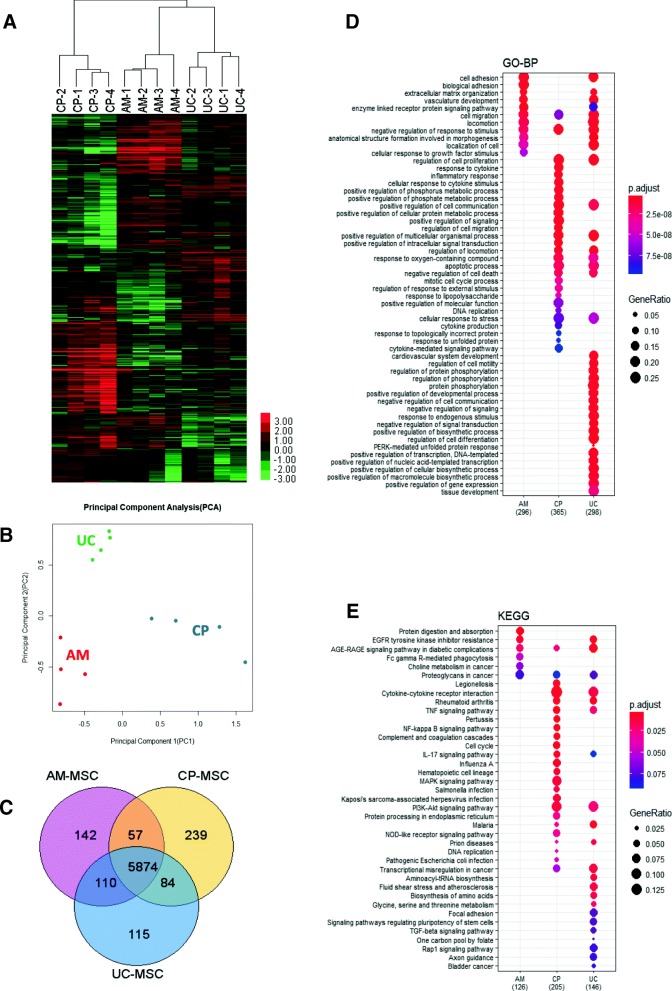
Transcriptional analysis of AM-MSCs, UC-MSCs, and CP-MSCs. Hierarchical clustering (**a**) and principal component analysis (**b**) showed a closer relationship between UC-MSCs and AM-MSCs, while CP-MSCs displayed a farther distance from UC-MSCs and AM-MSCs. **c** The number of differentially expressed genes between these three types of MSCs was shown on the Venn diagram. GO-BP (**d**) and KEGG analyses (**e**) were utilized to evaluate the functions of these three types of MSCs based on their differentially expressed genes

To investigate the transcriptional differences among these three MSCs expanded in SFM, four individual sample sets of AM-MSCs, UC-MSCs, and CP-MSCs at passage 5 were analyzed using RNA-Seq. The differentially expressed gene information and the relationship among these three MSCs were presented in the heat map (Fig. 5a) and the PCA image (Fig. 5b). It was found that each type of MSCs from four different donors could be clustered into the same group. UC-MSCs together with AM-MSCs could be clustered into a larger group, while CP-MSCs showed a little distant relationship from the former two.

To further understand the functional differences among the three types of MSCs, 142, 115, and 239 specifically expressed genes in AM-MSCs, UC-MSCs, and CP-MSCs were identified respectively (Fig. 5c and Additional file 3: Table S2). The expression patterns of these specifically expressed genes were illustrated and their functions were annotated based on the biological process (Fig. 5d) and signaling pathway (Fig. 5e) involved. Through the GO-BP analysis, it was suggested that AM-MSCs specifically expressed genes involved in biological adhesion. With respect to UC-MSCs, they specifically expressed genes related mainly to cardiovascular system development, cell motility, protein phosphorylation, cell communication, and biosynthetic process. CP-MSCs differentially upregulated genes involved mainly in response to cytokine, cytokine production, and inflammatory response (Fig. 5d), suggesting that CP-MSCs might display specific biological features such as immunological characteristics and cytokine secretion capability. It was also noticed that UC-MSCs specifically expressed genes involved in response to endogenous stimuli, while CP-MSCs highly expressed genes related to regulation of response to exogenous stimuli, in accordance with the biological functions of human placenta during the fetal development. Moreover, it took note that CP-MSCs specifically upregulated genes related to mitotic cell cycle process and DNA replication, consistent with the KEGG pathway analysis results (Fig. 5e), which together suggested that CP-MSCs possessed higher proliferation ability.

### CP-MSCs showed higher proliferation ability

**Fig. 6:**
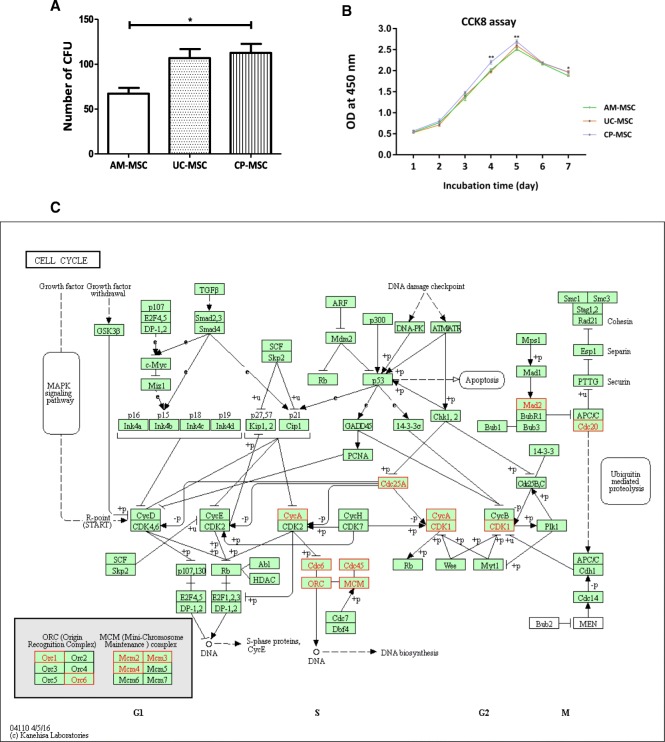
CP-MSC showed higher proliferation ability. The CFU (**a**) and CCK8 (**b**) assay of AM-MSCs, UC-MSCs, and CP-MSCs were shown. Among genes specifically expressed in CP-MSCs, those involved in the cell cycle pathway were marked in red and shown in (**c**). Namely, they are *Mad2*, *Cdc6*, *Cdc20*, *Cdc25A*, *Cdc45*, *CycA*, *CDK1*, *ORC1*, *ORC6*, *MCM2*, *MCM3*, and *MCM4*. All data were obtained from three independent donors and represented as the mean ± SEM (*n* = 3).*p < 0.05; **p < 0.01

To confirm the hypothesis that CP-MSCs might possess the higher proliferation ability, CFU test was performed to compare the proliferation capacity of these three neonatal MSCs. After the toluidine blue staining of cells, stained colonies were counted and then analyzed. The staining results clearly revealed that AM-MSCs exhibited the lowest proliferation ability among these three types of MSCs (see Additional file 4: Figure S2). Through the statistical analysis, it was indicated that CP-MSCs displayed significantly higher proliferation ability than AM-MSCs, while the proliferation ability differences between AM-MSCs and UC-MSCs were of no significance (Fig. 6a). In order to further confirm the higher proliferation ability of CP-MSCs, the three MSCs were then seeded at the same quantity for the CCK8 assay. The results revealed that the viable cell quantity among these three neonatal MSCs was almost the same at the first 3 days, but started to be significantly different from day 4 (Fig. 6b). The tendency of the growth curve once again verified our hypothesis that CP-MSCs possessed higher proliferation ability. Through the analysis of terms of CP-MSCs enriched in GO, we found that many of them were related to the positive regulation of cell proliferation or the negative regulation of cell death (see Additional file 5: Table S3). We then tried to dig further by analyzing CP-MSC specifically high-expressing genes involved in cell cycle pathway (Fig. 6c). We found that 12 genes in the cell cycle pathway were significantly higher expressed in CP-MSCs than in the other two neonatal MSCs. The higher expression of cyclin-dependent kinase gene (*CDK1*, as shown in Fig. 6c) could partly explain why CP-MSCs proliferated faster than the other two neonatal MSCs in SFM, considering its key role in the cell cycle [28]. Moreover, we found that many genes in Mini-Chromosome Maintenance (MCM) complex were also highly expressed in CP-MSCs. As an essential component of the pre-replication complex, MCM is of great importance for the initiation and elongation of DNA replication [29]. The higher expression of MCM might also promote the synthesis of DNA and then accelerate the proliferation of CP-MSCs in the SFM.

## Discussion

Human neonatal tissue-derived MSCs have been considered as promising candidates for cell therapy; however, comparative studies have indicated that MSCs of different neonatal origins or cultured under different conditions can exhibit different characteristics. In this study, AM-MSCs, UC-MSCs, and CP-MSCs were isolated and expanded in the chemical-defined commercial SFM, and their morphology immunophenotypes, trilineage differentiation potentials, and global gene expression patterns were systematically compared. The results showed that all these three MSCs exhibited typical MSC morphologies and immunophenotypic profiles, consistent with previous reported results in SCM [18]. As for the detailed trilineage differentiation efficiency, the results turned to be a little confusing. It was reported that the adipogenesis efficiency was higher in CP-MSCs than in UC-MSCs, whereas UC-MSCs exhibited more mineralized matrix accumulation than CP-MSCs in SCM [18], consistent with our results observed in SFM. It was also reported that MSCs of neonatal sources presented lower adipogenic ability but superior efficiency in osteogenesis in SCM [17]; however, when cultured in SFM, it was found that MSCs of different neonatal origins exhibited differently: the adipogenic ability of AM-MSCs was indeed very limited, while the ability of CP-MSCs and UC-MSCs was inspiring; AM-MSCs did show superior efficiency in osteogenic differentiation, whereas CP-MSCs could be hardly induced for osteogenesis. Taken together, our results indicated that MSC trilineage differentiation efficiency could be very different when cultured in SFM. As for the underlying molecular mechanisms, there are barely related researches reported yet. How can MSCs of different neonatal origins possess different trilineage differentiation potentials? To figure out this question, the transcriptome analysis of the three MSCs at serial differentiation points might be one direction remained for further work.

The global gene expression pattern analysis among MSCs derived from different neonatal tissues has been reported previously [15, 30]; however, as far as we know, there has been no comparative analysis of gene expression patterns among AM-MSCs, UC-MSCs, and CP-MSCs cultured in SFM reported yet. Considering that UC-MSCs displayed different gene expression patterns when cultured in SFM [21], our data first provided the differentially expressed gene information among these three neonatal MSCs cultured under the serum-free GMP condition. We were very interested that CP-MSCs specifically expressed genes involved in the mitotic cell cycle process and DNA replication, suggesting a stronger proliferation ability of CP-MSCs. Thus, CFU and CCK8 assay were performed and this speculation was finally validated. GO-BP results showed that CP-MSCs also specifically expressed genes related to the response to cytokine, cellular response to cytokine stimuli, cytokine production, and cytokine-mediated signal pathway, suggesting that placental CP-MSCs could potently secret cytokines and thus might exhibit some specific features, and we would like to take this as one of our future directions. Besides, some comparative studies reported before have discussed the heterogeneity of in vitro cultured MSCs. However, as for the deeper mechanisms whereby MSCs of different tissue origins or culture conditions could exhibit diverse biological features, they are left for more future work to unveil.

## Conclusions

In conclusion, our results presented the different trilineage differentiation potentials, gene expression patterns, and proliferation abilities among AM-MSCs, UC-MSCs, and CP-MSCs in SFM. To the best of our knowledge, this is the first systematic comparative work of MSCs from all these three neonatal tissues in the chemical-defined SFM. Our findings provide information and thus will contribute to the development of MSC-based cell therapy when identifying the optimal source of MSCs for a specific clinical application.

## Additional files

Additional file 1: **Table S1.** Primers used for real-time PCR. (PDF 466 kb)

Additional file 2: **Figure S1.** Immunophenotyping analysis of AM-MSCs, UC-MSCs, and CP-MSCs. (PDF 489 kb)

Additional file 3: **Table S2.** Differentially expressed gene. (TXT 109 kb)

Additional file 4: **Figure S2.** Toluidine blue staining results of the CFU test. (PDF 410 kb)

Additional file 5: **Table S3.** GO term enriched by genes specifically expressed in CP-MSCs (partly). (PDF 439 kb)

## Abbreviations

ADIPSIN: Complement factor D
AM: Amniotic membrane
AM-MSC: Amniotic membrane-derived MSC
ANOVA: Analysis of variance
CFU: Colony-forming unit
COL2A1: Collagen type II alpha 1 chain
COMP: Cartilage oligomeric matrix protein
CP: Chorionic plate
CP-MSC: Chorionic plate-derived MSC
CV: Chorionic villi
DCN: Decorin
DPBS: Dulbecco’s phosphate-buffered saline
FITC: Fluorescein isothiocyanate
FPKM: Fragments per kilobase million
GMP: Good Manufacturing Practice
GO-BP: Gene ontology–biological process
HLA: Human leukocyte antigen
ISCT: International Society for Cellular Therapies
KEGG: Kyoto Encyclopedia of Genes and Genomes
LEP: Leptin
MHC: Major histocompatibility complex
MSC: Mesenchymal stem cell
OCN: Bone gamma-carboxyglutamate protein
ON: Secreted protein acidic and cysteine rich
PCA: Principal component analysis
PE: Phycoerythrin
PFA: Paraformaldehyde
PPARG: Peroxisome proliferator activated reporter gamma
RUNX2: Runt-related transcription factor 2
SCM: Serum-containing medium
SEM: Standard error of mean
SFM: Serum-free medium
UC: Umbilical cord
UC-MSC: Umbilical cord-derived MSC
