# Supplementary material for: Comparative analysis of mesenchymal stem cells derived from amniotic membrane, umbilical cord, and chorionic plate under serum-free condition
Source: Stem Cell Res Ther. 2019 Jan 11;10:19. doi: 10.1186/s13287-018-1104-x (PMC6330472; doi:10.1186/s13287-018-1104-x)
Supplement: Supplementary file 1 — Table S1. Primers used for real-time PCR. (PDF 466 kb) [file 13287_2018_1104_MOESM1_ESM.pdf]

Table S1 Primers used for real-time PCR

	Forward sequence (5'-3')	Reverse sequence (5'-3')
PPARG	ACCAAAGTGCAATCAAAGTGGA	ATGAGGGAGTTGGAAGGCTCT
LEP	TGCCTTCCAGAAACGTGATCC	CTCTGTGGAGTAGCCTGAAGC
ADIPSIN	GACACCATCGACCACGACC	GCCACGTCGCAGAGAGTTC
OCN	GGCGCTACCTGTATCAATGG	GTGGTCAGCCAACTCGTCA
RUNX2	CCGCCTCAGTGATTTAGGGC	GGGTCTGTAATCTGACTCTGTCC
ON	TGAGGTATCTGTGGGAGCTAATC	CCTTGCCGTGTTTGCAGTG
COL2A1	TGGACGATCAGGCGAAACC	GCTGCGGATGCTCTCAATCT
COMP	CGAGTCCGCTGTATCAACACC	TCCGTGCAAACCTGCTTGT
DCN	ATGAAGGCCACTATCATCCTCC	GTCGCGGTCATCAGGAACTT
ACTB	TGGCACCCAGCACAATGAA	CTAAGTCATAGTCCGCCTAGAAGA

Abbreviations: PPARG, peroxisome proliferator activated receptor gamma; LEP, leptin; LPL, lipoprotein lipase; OCN, bone gamma-carboxyglutamate protein; RUNX2, runt-related transcription factor 2; ON, secreted protein acidic and cysteine rich; COL2A1, collagen type II alpha 1 chain; BGN, biglycan; DCN, decorin; ACTB, actin beta.
