# Supplementary material for: Comparative analysis of mesenchymal stem cells derived from amniotic membrane, umbilical cord, and chorionic plate under serum-free condition
Source: Stem Cell Res Ther. 2019 Jan 11;10:19. doi: 10.1186/s13287-018-1104-x (PMC6330472; doi:10.1186/s13287-018-1104-x)
Supplement: Supplementary file 2 — Figure S1. Immunophenotyping analysis of AM-MSCs, UC-MSCs, and CP-MSCs. (PDF 489 kb) [file 13287_2018_1104_MOESM2_ESM.pdf]

Figure S1 Immunophenotyping Analysis of AM-MSCs, UC-MSCs and CP-MSCs.

Antibodies (%)	AM-MSCs	UC-MSCs	CP-MSCs
CD73	99.54 ± 0.13	99.95 ± 0.02	99.52 ± 0.14
CD90	99.98 ± 0.02	100.00 ± 0.00	99.97 ± 0.03
CD105	99.82 ± 0.08	99.99 ± 0.01	99.35 ± 0.25
HLA-DR	0.08 ± 0.08	0.03 ± 0.02	0.03 ± 0.03
CD14	0.28 ± 0.06	0.04 ± 0.02	0.47 ± 0.24
CD19	0.04 ± 0.03	0.00 ± 0.00	0.05 ± 0.03
CD34	0.34 ± 0.33	0.14 ± 0.08	0.19 ± 0.10
CD45	0.21 ± 0.10	0.12 ± 0.02	0.41 ± 0.07

Immunophenotyping analysis of MSCs derived from three individual donors. All data were presented as the mean ± SEM (n = 3).
