# Supplementary material for: Comparative analysis of mesenchymal stem cells derived from amniotic membrane, umbilical cord, and chorionic plate under serum-free condition
Source: Stem Cell Res Ther. 2019 Jan 11;10:19. doi: 10.1186/s13287-018-1104-x (PMC6330472; doi:10.1186/s13287-018-1104-x)
Supplement: Supplementary file 4 — Figure S2. Toluidine blue staining results of the CFU test. (PDF 410 kb) [file 13287_2018_1104_MOESM4_ESM.pdf]

Figure S2 Toluidine blue staining results of the CFU test

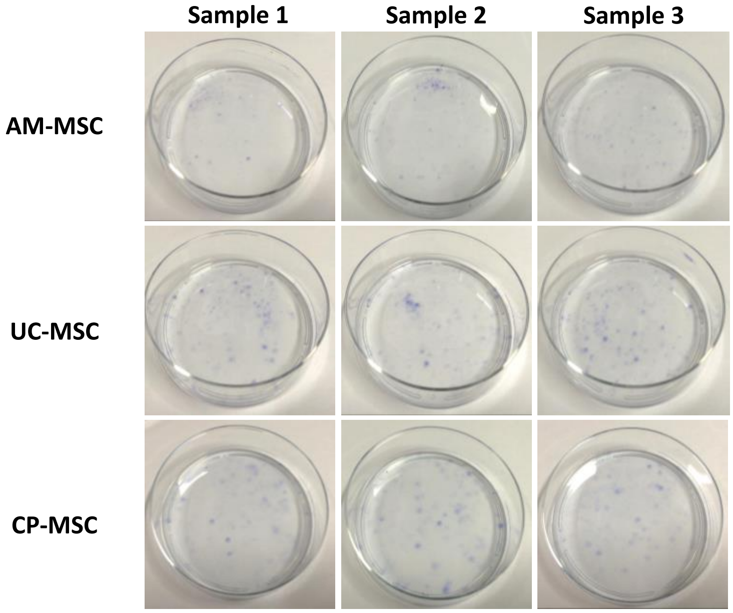

Toluidine blue staining photos of the CFU test. AM-MSC, UC-MSC and CP-MSC samples from three individual donors were tested .
