# Supplementary material for: Comparative analysis of mesenchymal stem cells derived from amniotic membrane, umbilical cord, and chorionic plate under serum-free condition
Source: Stem Cell Res Ther. 2019 Jan 11;10:19. doi: 10.1186/s13287-018-1104-x (PMC6330472; doi:10.1186/s13287-018-1104-x)
Supplement: Supplementary file 5 — Table S3. GO term enriched by genes specifically expressed in CP-MSCs (partly). (PDF 439 kb) [file 13287_2018_1104_MOESM5_ESM.pdf]

Table S3 Gene Ontology (GO) term enriched by genes specifically expressed in CP-MSCs (partly)

GO term	Count
GO:0042127 regulation of cell proliferation	86
GO:0008284 positive regulation of cell proliferation	56
GO:0060548 negative regulation of cell death	54
GO:1903047 mitotic cell cycle process	49
GO:0043066 negative regulation of apoptotic process	50
GO:0043069 negative regulation of programmed cell death	50
GO:0006260 DNA replication	26
GO:0022402 cell cycle process	65

GO term enriched by CP-MSC specifically expressed genes was partly presented. Through these terms, we could see the positive regulation of cell proliferation together with the negative regulation of cell death in CP-MSCs, which might be an explanation for the higher proliferation ability of CP-MSC.
